# Epigenetics and type II diabetes mellitus: underlying mechanisms of prenatal predisposition

**DOI:** 10.3389/fcell.2014.00015

**Published:** 2014-05-05

**Authors:** J. David Sterns, Colin B. Smith, John R. Steele, Kimberly L. Stevenson, G. Ian Gallicano

**Affiliations:** Department of Biochemistry and Molecular and Cellular Biology, Georgetown University School of MedicineWashington, DC, USA

**Keywords:** epigenetics, type II diabetes mellitus, DNA methylation, histone modifications, intrauterine development

## Abstract

Type II diabetes mellitus (T2DM) is a widespread metabolic disorder characterized by insulin resistance precipitating abnormally high blood glucose levels. While the onset of T2DM is known to be the consequence of a multifactorial interplay with a strong genetic component, emerging research has demonstrated the additional role of a variety of epigenetic mechanisms in the development of this disorder. Heritable epigenetic modifications, such as DNA methylation and histone modifications, play a vital role in many important cellular processes, including pancreatic cellular differentiation and maintenance of normal β-cell function. Recent studies have found possible epigenetic mechanisms to explain observed risk factors, such as altered atherogenic lipid profiles, elevated body mass index (BMI), and impaired glucose tolerance (IGT), for later development of T2DM in children born to mothers experiencing both famine and hyperglycemic conditions. It is suggested that these epigenetic influences happen early during gestation and are less susceptible to the effects of postnatal environmental modification as was previously thought, highlighting the importance of early preventative measures in minimizing the global burden of T2DM.

## Introduction

The worldwide rise in type II diabetes mellitus (T2DM) is a public health issue that must be addressed. According to the International Diabetes Federation, there were 366 million people living with diabetes in 2011, and this number is expected to rise to 552 million by 2030 (Whiting et al., 2011). T2DM is a chronic and largely preventable disease defined by high blood glucose resulting from insulin resistance and is characterized by risk factors such as family history of disease, obesity, high blood pressure, and high low-density lipoprotein (LDL) and low high-density lipoprotein (HDL) levels (American Diabetes Association, 2013). Recent research has shown that fetal development within a mother's uterus is affected by the mother's nutritional intake and lifestyle, both of which can greatly increase the risk of the fetus developing T2DM much later in life. Epigenetics, or the heritable changes in gene expression that occur without changes in the primary DNA sequence, has recently been proposed as a route through which this process occurs (Russell, 2010).

The main mechanisms through which epigenetics acts to affect cell phenotype and biological processes are DNA methylation and histone modifications (Bramswig and Kaestner, 2012). DNA methylation involves DNA methyltransferases modifying cytosines to create 5-methylcytosine, with the majority of this action occurring in CpG islands found in multiple protein-coding gene promoter regions throughout the human genome. Methylation of cytosines in these areas is associated with repression of transcription (Russell, 2010). This gene silencing occurs through direction prevention of transcriptional promoter binding as well as the recruitment of transcriptional repressors (Bramswig and Kaestner, 2012). Repressors that bind to methylated CpG islands then initiate the cascade that ultimately results in the second primary mechanism of epigenetic regulation: histone modifications and the recruitment of histone deacetyltransferases (HDACs) (Russell, 2010).

As Russell notes, there are many specific means by which a histone is modified, including methylation, phosphorylation, or ubiquitination at varying locations within the complex. However, one of the most extensively studied is that of acetylation and deacetylation of the histone tails, a delicate balance that serves to alter the charge of the histone core, ultimately determining the ease by which DNA transcription can occur. For example, HDACs are capable of deacetylating amino-terminal lysine residues on histone tails, permitting the positively charged lysine residues of the histone to bind more tightly to the negatively-charged DNA wrapped around it. This tighter binding allows for more compact wrapping around the structural scaffolding provided by the histones. Genes located within this area of tighter binding are, therefore, transcriptionally repressed as the transcription machinery is unable to access the gene promoter (2010).

These epigenetic alterations, of which much is known through various studies *in vitro*, have also been shown *in vivo* to be associated with environmental influences, not least of which is the intrauterine environment and its impact on a developing fetus (Bramswig and Kaestner, 2012). Here, we focus on this environmental link, its epigenetic impact, and the molecular implications of those epigenetic alterations as points of further exploration by researchers and novel therapeutic intervention by clinicians seeking to proactively care for patients at risk for T2DM.

## Prenatal nutrition and its role in the development of T2DM in offspring

A current focus in the field of epigenetics is the exploration of metabolic changes within the intrauterine environment and their implication on the development of risk factors associated with T2DM postnatally. The impact of maternal nutrition on lipid profiles, impaired glucose handling, high blood pressure, and obesity, among other risk factors for T2DM, are areas of interest at this time. With this in mind, the Dutch famine from 1944 to 1945 and the great amount of data collected from individuals who felt its effects firsthand offers a unique study cohort to delve into this topic, with a very brief shortage of food lasting only 5 months juxtaposed against a backdrop of adequate nutrition before and after. Additionally, the short time period over which the starvation conditions were experienced inevitably creates for interesting study parameters in which pregnant mothers were exposed to these harsh conditions at different trimesters in their pregnancy (Roseboom et al., 2000). Meticulous records and census data spanning several decades reveal the far-reaching impact that prenatal diet during different trimesters has on the offspring's health in adulthood. Furthermore, what is even more astonishing is the discovery that these impacts are observed even into subsequent generations (Hillier et al., 2007; Ding et al., 2012).

### Prenatal exposure to famine and lipid profiles in adulthood

A clear relationship has been found between exposure to famine *in utero* and the development of an atherogenic lipid profile as an adult. An atherogenic lipid profile is defined as high LDL levels in conjunction with low HDL levels, or having a high LDL:HDL ratio (Roseboom et al., 2000). High LDL levels are recognized by the American Diabetes Association as a major risk factor for the development of T2DM (American Diabetes Association, 2013). Painter et al. found that fetuses exposed to famine at any point during gestation had elevated LDL:HDL ratios as adults compared to persons not exposed (2006). Within this group, it seems that persons exposed to famine early in their mothers' pregnancy rather than mid- to late-gestation are at a further increased risk for developing an atherogenic lipid profile, although women had a higher atherogenic lipid profile than men (Roseboom et al., 2000).

Interestingly, a more recent look into the same Dutch famine data found only women to have significantly increased lipid profiles, an assessment of total cholesterol, LDL, and HDL. This discrepancy may be due to the fact that earlier studies neglected to look at men and women groups separately in their analyses and also did not include sibling controls. Taking this into account, Lumey et al. compared two genetically-similar individuals and gained a more accurate perspective of the direct effects of famine on the fetus. Based on these findings, it is thought that prenatal exposure to famine may have sex-specific outcomes (Lumey et al., 2009). They further postulate that these outcomes may be due to separate controls of metabolic pathways *in utero*; this view is supported by Roseboom et al., who suggest that there may be specific times during gestation when there is a strong potential to influence glucose and cholesterol metabolism (Roseboom et al., 2000; Lumey et al., 2009). Regardless of when it occurs and to whom, it is clear that prenatal exposure to famine during gestation increases the risk of developing an atherogenic lipid profile later in life, possibly through epigenetic mechanisms, thereby increasing the risk for the person developing T2DM.

### Prenatal exposure to famine/overfeeding and the risk of obesity in adulthood

Ravelli et al. found that exposure to famine in the first half of pregnancy increased the risk of developing obesity as adults, yet an increased BMI was only found in persons exposed very early in gestation, mirroring the trend found with lipid profiles. These results suggest that controlling mechanisms for obesity must be separate from those influencing BMI (Ravelli et al., 1998). While further work is needed to determine the reasons why varying exposures lead to these differences, it is quite apparent that exposure to famine during pregnancy leads to an overall increased risk for obesity and increased BMI as adults, both of which are important risk factors for the development of T2DM.

Furthermore, a cohort study carried out on 513,501 women from 1989 to 2003 found that maternal weight gain throughout pregnancy increases the birthweight of children independently of genetic factors (Ludwig and Currie, 2010). Much of this arises from the physiological response of the fetus to overnutrition, with normal maternal insulin resistance associated with pregnancy becoming exaggerated in obese states and increasing the shunting of vital nutrients to the growing fetus. The fetus, in turn, secretes its own insulin in response, increasing glucose uptake, and fat storage, and thus causing the increased birth weights of babies born under these conditions (Ludwig and Currie, 2010). This effect on the developing fetus carries over even into adulthood, presumably through epigenetic changes pre-natally, with the mother's fasting hyperglycemia providing one of the most profound and well-studied associations with immediate and long-term childhood obesity risk (Hillier et al., 2007).

The means by which this occurs is further complicated by the existence of so called endocrine-disrupting chemicals (EDCs) that have the capacity to influence most if not all aspects of adipose tissue growth, from undifferentiated stromal cells to mature adipocytes, a phenomenon that is especially detrimental to children and young adults who are physiologically in the midst of a time of intense adipocyte hyperplasia (Janesick and Blumberg, 2011). As Janesick and Blumberg note, these EDCs, including a wide range of both natural and man-made substances such as pharmaceuticals, pesticides, and plasticizers like bisphenol A, possess the ability to modulate important cellular-signaling pathways via epigenetic alterations (2011). Whether through normal physiological responses to extraordinary circumstances or by exogenous substances interacting with the fetal epigenome, it is clear that there is much more at stake here. When it comes to pregnancy, healthcare personnel, mothers, and society at large must be aware that any deviation from ideal fetal development conditions will have implications on both immediate maternal and fetal health as well as on the long-term wellness of the child as he or she moves into adulthood.

### Prenatal exposure to famine and risk of coronary artery disease in adulthood

While coronary artery disease (CAD) itself is not a direct risk factor for T2DM, CAD is associated with high blood pressure, which is an identified risk factor for the development of T2DM. Therefore, it is not uncommon for a person with T2DM to also suffer from CAD as the two diseases are intimately linked through this association. As such, the development of CAD offers yet another glimpse into a individual's underlying susceptibility to the development of T2DM. An increased rate of CAD in offspring was found to be positively related to maternal exposure to famine while pregnant (Painter et al., 2006). Similar to the trends seen with lipid profiles and BMI, persons exposed to famine early in pregnancy were more likely to develop CAD as adults than those exposed in mid- to late-pregnancy, with a mean age of diagnosis at 47 years of age for those exposed early during the first trimester and a mean age of diagnosis at 50 years for those exposed later in the developmental process. Interestingly, persons exposed in mid- to late-pregnancy did not have a significantly increased risk for developing CAD than the control group (Painter et al., 2006). This has interesting implications on the development of T2DM, as these findings would indicate that there are molecular events that occur specifically early in gestation that are highly susceptible to the state of maternal health and nutrition.

### Prenatal exposure to famine and impaired glucose tolerance in adulthood

While exposure to famine early in gestation seems to have the largest impact on future lipid profiles and the development of CAD, it was found that mid- to late-exposure to famine *in utero* appears to have the largest effect on glucose tolerance (Ravelli et al., 1998). Adults exposed to famine at any point during gestation had higher rates of IGT compared to controls, but persons exposed in mid- to late-pregnancy had the highest rates of IGT or T2DM (Ravelli et al., 1998; Painter et al., 2006). Furthermore, the persons with the greatest level of glucose intolerance were the most likely to be obese. It is important to note that IGT was found in adults of all body types with prenatal exposure, suggesting that the underlying mechanism for IGT was not secondary to obesity but, rather, the cause of it (Ravelli et al., 1998).

These results further solidify earlier explorations of a potential association between events experienced prenatally in varying intrauterine environments and the significant effects that exposure has on metabolic control systems. Wellen et al. went a step further to piece together the missing link between the overlying epigenetic imprint evidenced by histone acetylation and the means by which this epigenetic influence establishes itself in concordance with environmental change. By measuring global histone acetylation levels in various metabolic states, her group was able to show that ATP-citrate lyase (ACL), a metabolically important enzyme critical in the conversion of glucose-derived citrate into acetyl-CoA, was needed for histone acetylation. Interestingly, this acetylation coincided with times of plentiful glucose supply, tipping the balance to favor global histone acetylation over HDAC-induced deacetylation, allowing for the expression of specific metabolic genes as well as promote cellular differentiation into adipocytes (Wellen et al., 2009). As glucose intolerance and obesity are major hallmarks of T2DM, this insight is promising for further elucidation of epigenetic influence on future development of chronic disease, in general, and T2DM, specifically.

### Timing of prenatal exposure to famine/overfeeding and its effect on methylation

What has been a source of confusion is the complex timing by which epigenetic mechanisms exert their influence on DNA transcription. For example, the specific regulatory action on the differentially methylated region (DMR) of the insulin-like growth factor II (*IGF2*) and *H19* promoters early in gestation may have long-term effects and may not be susceptible to further epigenetic modification via environmental or age-related stimuli as was previously thought (Heijmans et al., 2007). In a study comparing the extent of methylation at specific CpG islands in human adolescent and middle aged twins, it was found that no significant distinguishing epigenetic marks could be attributed to differences in age at these particular loci but, instead, revealed an astonishing consistency in the methylation status, once methylated, of the *IGF2* and *H19* promoters (Heijmans et al., 2007). These findings challenge other studies that suggested the possibility of global epigenetic pliancy arising through the influence of environment, diet, and age (Fraga et al., 2005). A more recent look into the epigenetic footprint of DNA taken from a cohort of monozygotic twins goes one step further by suggesting that DNA promoter regions with the greatest level of regulatory action retained the most stable methylation patterns while those loci that were considered not as important functionally showed a greater level of variability in epigenetic imprinting (Kaminsky et al., 2009). With this discovery, epigenetic influence on a global level is now believed to occur prior to birth with more subtle changes to loci of lesser significance occurring throughout life (Kaminsky et al., 2009).

Heijmans et al. argue that both prenatal and postnatal epigenetic mechanisms offer plausible explanations for the ultimate expression of genetic information, but it appears that, at least in the cases of these particular loci, global epigenetic variability in methylation elsewhere in the genome as a result of any number of environmental factors has a more temporal and, thus, more limited impact on ultimate phenotypic expression when compared to the effects of stable methylation patterns established in the embryo at *IGF2* and *H19* in response to intrauterine environment. Therefore, they propose that epigenetic modifications occurring during embryological development tend to exert a greater phenotypic influence on the organism and are more stable in relation to the length of their influence (Heijmans et al., 2007). Intuitively, such an early alteration to genetic expression in cells of a developing fetus carries with it enormous implications for the cells that are to arise after subsequent mitotic events. By changing the course of an individual cell, especially a stem cell or an early progenitor, epigenetics can act on a scale that for all intents and purposes is global and widespread while at the same time possessing the ability to exert a more tissue specific influence if the effect occurs after the onset of important differentiation events well into an individual's life (Kirchner et al., 2013).

Notably, varying methylation patterns at this same *IGF2* locus were also observed in adults exposed *in utero* to the Dutch famine depending upon the timing of their prenatal exposure. Individuals who were conceived before the German-imposed famine and, thus, exposed to the associated effects of malnutrition on the mother later in their gestation period had similar methylation patterns to those who were not exposed to famine at all. However, those who were conceived in the midst of the famine saw an ensuing hypomethylation at CpG islands within this important promoter site and was readily observed even 60 years later at the time data from the cohort study was originally released (Heijmans et al., 2008). Although this is not exactly correlative with the hypermethylation observed in offspring born to GDM mothers, the study suggests a time of critical importance early in embryological development for epigenetic influence with the potential for lasting regulatory action on this specific portion of the human genome.

The results found from studying the effect of prenatal nutrition on lipid profiles, obesity, and glucose handling have interesting implications for the development of T2DM in adults. These are well-appreciated risk factors for T2DM, and exposure to famine during pregnancy was found to increase all of these risk factors. However, the timing of exposure to famine and the effect it has, overall, on fetal metabolic development is less clear. Highest increased rates of atherogenic lipid profiles, obesity, and CAD were found with early exposure, while highest increased rates of glucose intolerance and T2DM itself were found in mid- to late-exposure. Studies conducted after the publishing of the glucose intolerance results expressed contrasting perspectives and highlighted that exposure early in gestation, specifically the first trimester, was most significant for future risk (Heijmans et al., 2008). In conjunction with other recent studies that have stressed the importance of early exposure to adverse events *in utero*, the emphasis that Ravelli et al. places on the correlation between late-gestational exposure and increased risk for future development of IGT and T2DM is questionable (Heijmans et al., 2008; Ding et al., 2012).

Given the recent findings and discrepancies in current studies discussed earlier, more work is needed to determine the influence that timing of gestational exposure to various intrauterine insults has on future T2DM development. Once this is achieved, it will be far less difficult to clarify the means by which prenatal epigenetic imprinting interplays with the enormous epigenetic variation induced postnatally by various environmental stimuli and aging, pharmaceutical intervention, and lifestyle choices such as exercise (Fraga et al., 2005; Kaminsky et al., 2009; Pinney et al., 2011; Nitert et al., 2012; Kirchner et al., 2013). Indeed, as others have noted previously, such an integration must also incorporate information regarding more conventional regulators of gene transcription known to play a pivotal role in immediate cellular response to changing environmental stimuli. This is no small task, especially considering the vast unknown that the realm of epigenetics still poses as well as the substantial ethical obstacles to studying these alterations in humans, but by delving ever deeper in this particular field, science will be one step ever closer to understanding the true mechanisms by which organisms respond, survive, and thrive in an incredibly complex and dynamic world (McGraw et al., 2013).

## Other genes believed to be implicated in the development of T2DM

Tobi et al., working off previous studies that had shown hypomethylation of the *IGF2* DMR to be a result of exposure to famine during gestation, decided to look at the patterns of 15 other possible genes implicated in metabolic and cardiovascular disease in 60 people conceived during the Dutch famine. Changes in methylation were seen in six of these 15 candidate genes in people conceived during the famine compared to their siblings. Hypermethylation was observed in the *GNASAS, MEG3, IL10, ABCA1*, and *LEP* proximal promoters; hypomethylation was observed in the INSIGF promoter. When compared to individuals that were exposed later in gestation, methylation changes in *IL10, GNASAS*, and *INSIGF* were found to be time-sensitive. Also, alteration of the methylation status in *INSIGF, GNASAS*, and *LEP* were sex-specific with methylation at *GNASAS* and *LEP* being specific for men (Tobi et al., 2009). These results support previous findings that exposure to famine has timing- and sex-specific effects on a developing embryo.

## Stem cells: unlocking the epigenetic pattern

A recent study by Xie et al. looking into the differentiation of human embryonic stem cells (hESCs) into pancreatic β cells proposes a strong epigenetic influence involving chromatin remodeling Polycomb group (PcG) proteins. PcG-dependent repression involves a bivalent histone trimethylation of the H3K4 and H3K27 histones at particular gene loci often encoding important developmental regulatory proteins. This repression renders these genes inaccessible and transcriptionally inactive. However, when trimethylation at H3K27 is removed and the PcG-mediated repression of that gene is released, that particular locus is primed for transcription. Many genes encoding transcription factors necessary for early pancreatic development, including *SOX9*, *PDX1*, *PTF1A*, *HNF6*, *NK6.1*, and *NK6.2*, have been shown to be regulated in this manner (Xie et al., 2013). This implies a significant role for epigenetic mechanisms early in the regulation of stem cell differentiation into pancreatic endocrine cells.

Recently, this epigenetic interaction has been suggested to exert its effects even earlier, by presenting a likely regulatory influence on the onset of embryonic genome activation (EGA) prior to implantation (Vassena et al., 2011). During the first few cycles of cellular mitosis post-fertilization, the embryo relies primarily on mRNA and protein inherited from the mother in order to carry out the rapid division and high metabolic activity characteristic of this stage of development. These mechanisms involve an initial translation of maternal mRNA and catabolism of oocyte-inherited proteins in the two-cell stage before proper initiation of embryonic is able to occur (Wang and Latham, 2000; Bushati et al., 2008). Interestingly, this pre-EGA translation of maternal mRNA involves two waves of activity, the first involving the translation of protein products that persist throughout this critical stage of development, suggesting that they have important regulatory roles in the initiation of EGA, while the second wave is highlighted by protein breakdown in order to feed the rapidly developing cells (Vassena et al., 2011). This process is both dynamic and occurs before any fetal genomic expression has taken place, with epigenetic-induced polyadenylation of maternal mRNA suggested as a possible regulatory mechanism to help explain the occurrence of these waves of maternal mRNA translation (Seydoux, 1996; Vassena et al., 2011).

Vassena et al. also found *POU5F1*, a critical regulator in cellular pluripotency, to be involved in these pre-EGA epigenetic events. One of the first genes transcribed by the fetal genome, *POU5F1* is expressed even before EGA occurs through a recombination of maternally-inherited mRNA up until the 2-cell stage. This process is only relieved by the activation of fetal genomic transcription, which carries out the transcription of this important regulator for the remainder of embryonic development. Once this occurs, other pluripotency genes such as *NANOG*, *SOX2*, *KLF4*, *and ZFP42*, begin to be expressed in a sequential manner, hinting at a hierarchical scheme by which pluripotency is acquired in a developing human embryo with *POU5F1* playing the role of master regulator which, when properly expressed, paves the way for the expression of other pluripotency genes (Vassena et al., 2011). This work deals primarily with discovering additional avenues, specifically in altering the epigenome of cells already differentiated, from which to circumvent the ethical obstacles imposed on research using pluripotent cells. However, this early epigenetic influence on genomic activation could 1 day prove to have ramifications not only for somatic cells reprogramming but for more targeted and preventative epigenetic therapeutic interventions as well.

Similarly, epigenetics can also influence the developmental process later in the fetal development timeline, as seen in already-differentiated β cells. Current exploration by Ding et al. suggests a relationship between T2DM and the insulin-like growth factor II *IGF2* and H19 fetal liver mRNA (*H19*) genes, both of which encode for fundamental proteins involved in normal pancreatic â-cell function. It was found that under certain conditions, such as intrauterine hyperglycemia typical to gestational diabetes mellitus (GDM), these genes are epigenetically downregulated through the addition of methyl groups at CpG islands within highly variable DMRs of their promoter regions (Ding et al., 2012). The hypermethylation of these imprinted genes *IGF2*, which is maternally methylated, and *H19*, which is paternally methylated, corresponded to a decrease in the mRNA expression and subsequent protein product translation, a swollen and disordered endoplasmic reticulum, as well as an increased insulin response during feeding and decreased fasting insulin levels within pancreatic â-cells isolated from mice offspring of GDM mothers when compared to control offspring (Ding et al., 2012). This provides strong evidence to a possible embryological epigenetic component to future development of insulin desensitivity associated with T2DM later in life. Furthermore, inhibited *IGF2* and *H19* gene expression was also observed in the sperm of adult males of GDM mothers, suggesting that intrauterine environment epigenetically impacts not only somatic cells but germ cells as well, explaining its potential for transgenerational transmission (Ding et al., 2012).

In essence, a key reason why no current stem cell, albeit embryonic, adult, or induced pluripotent stem cells, can generate the levels of insulin needed to cure diabetes in mice is most likely due to lack of proper epigenetic regulation as highlighted by the mechanisms mentioned earlier. Correcting epigenetic regulation at the stem cell level *in vitro* could be the answer to proactive treatment, and possibly cure, of Type 1 diabetes mellitus (T1DM) and T2DM.

## Prenatal epigenetic regulation and its lasting effect on epigenetic regulation postnatally

New insights into both a prenatal and postnatal epigenetic influence on the same gene locus are now being expressed. One study performed by Park et al. looked at *PDX1*, which, like *IGF2* and *H19*, plays an essential role in the regulation of β-cell growth, function, and production of insulin. Their group discovered that decreased expression of *PDX1* was shown to contribute to the progression of T2DM in offspring that experienced intrauterine growth retardation (IUGR) during embryological development. As a result of limited supply of oxygen and other nutrients in the intrauterine environment, IUGR fetal islet cell H3 and H4 histones associated with the *PDX1* promoter exhibit significant decreases in acetylation by means of HDAC recruitment (Park et al., 2008). As noted previously, it is an interaction between a hyperacetylated H4 in the region of the insulin gene promotor and *PDX1* that allows for glucose-mediated production of insulin in β-cells. Without this histone hyperacetylation as observed in low-glucose environments, β-cell metabolic function is impaired (Ling and Groop, 2009).

This nutritional deficiency alone is enough to cause decreased levels of *PDX1* protein product, but the effect is further compounded with the progression of age postnatally via increased deacetylation of the core histones H3 and H4 and a decreased trimethylation on histone 3 lysine 4 (H3K4) along with a subsequent dimethylation on histone 3 lysine 9 (H3K9), both of which contribute to the inhibition of genetic expression at this locus (Park et al., 2008). This was thought to be caused by HDAC inducement of a demethylase-catalyzed demethylation of H3K4, which is normally methylated and responsible for the maintenance of an active chromatin state, thereby providing the opportunity for potential methylation of H3K9 and the switch to an inactive chromatin state (Park et al., 2008).

As Park et al. suggest, the heightened H3K9 methylation by histone methyltransferases concurrent with the passing of time and postnatal exposure to certain environmental factors promotes DNA methylation of CpG islands in the *PDX1* promoter, providing yet another channel for inhibitory regulation as discussed earlier (2008). These modifications to the histone structure and alteration of the overall DNA conformation result in increasingly restricted access for the critical activator protein USF-1 to the promoter, inhibiting *PDX1* transcription and, eventually, provoking the glucose homeostatic mismanagement accompanying β-cell dysfunction that eventually manifests as T2DM (Park et al., 2008). What is interesting is that recent mice study results from Pinney et al. indicate that this aberrant epigenetic repression, one that arises as a survival response to harsh intrauterine conditions experienced in IUGR, can be reversed by a short course treatment of Exendin-4, a glucagon-like peptide-1 analog. It is thought that this occurs by negating the effects of decreased phosphorylation and activation of USF-1 under IUGR conditions via activation of a signaling cascade culminating in the phosphorylation of many important pancreatic beta cell proteins, allowing for proper USF-1 binding to the *PDX1* promoter region and the subsequent transcription of the all-important *PDX1* protein product (Pinney et al., 2011).

In summary, *PDX1* plays a critical role in the development of the pancreatic β-cell as well as maintaining its proper function. As Pinney and Simmons point out, if expression of *PDX1* is completely inhibited, either by mutation or regulatory mechanisms highlighted above, pancreatic agenesis is often the end-result, offering a possible explanation for the early onset of the T1DM phenotype. However, if expression is maintained at a minimal level, normal β-cell mass can be achieved but with concordant dysfunction in glucose-stimulated insulin secretion, eventually resulting in T2DM (Pinney and Simmons, 2010).

## Conclusion

**Figure 1:**
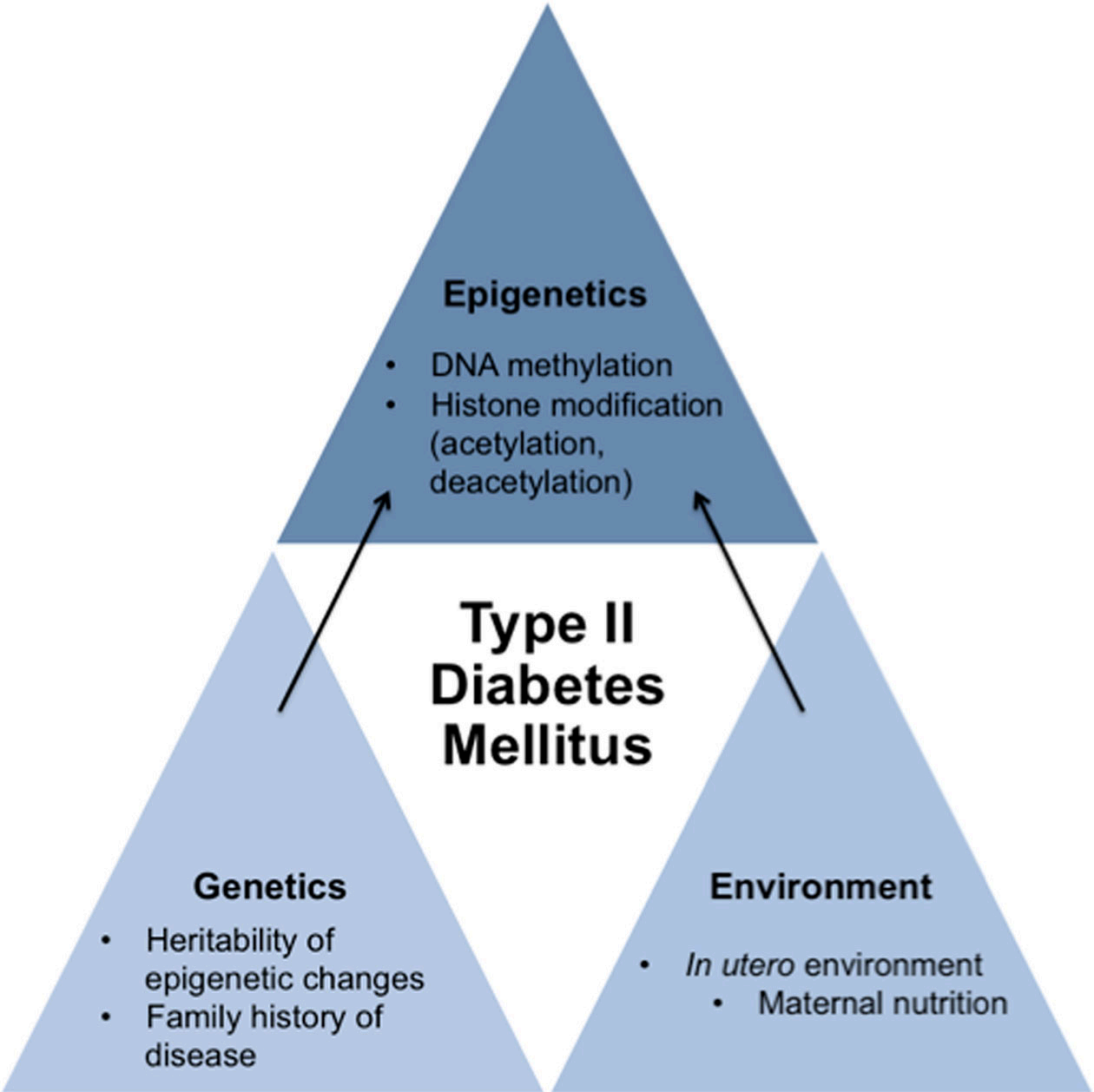
**Schematic proposing how genetics, environment, and epigenetics play a role in contributing to the development of type II diabetes mellitus**. While genetics and environment have an impact on the development of T2DM on their own, new research indicates that *in utero* environment may cause specific epigenetic changes to the fetus's genome that may cause susceptibility of the development of T2DM of the fetus later in life. Therefore, epigenetics also has an impact on the development of T2DM.

Although it is widely known that genetics and lifestyle have a profound impact on the development of T2DM, recent research surrounding epigenetics has shown that exposure of the fetus to various abnormal intrauterine environmental states like hyperglycemia and famine can also increase the risk of developing T2DM. Since methylation of CpG islands may be heritable, epigenetic activity occurring in the developing fetal genome may result in lasting effects on our metabolic control. These lasting effects most likely introduce critical factors in the development of T2DM later in life (Hillier et al., 2007; Park et al., 2008; Ding et al., 2012). Thus, the influence that epigenetic mechanisms exert may be immensely important not only as a means by which environmental factors impact development of T2DM but also in its role in establishing one's risk profile for developing T2DM even before birth (Figure 1).

**Figure 2:**
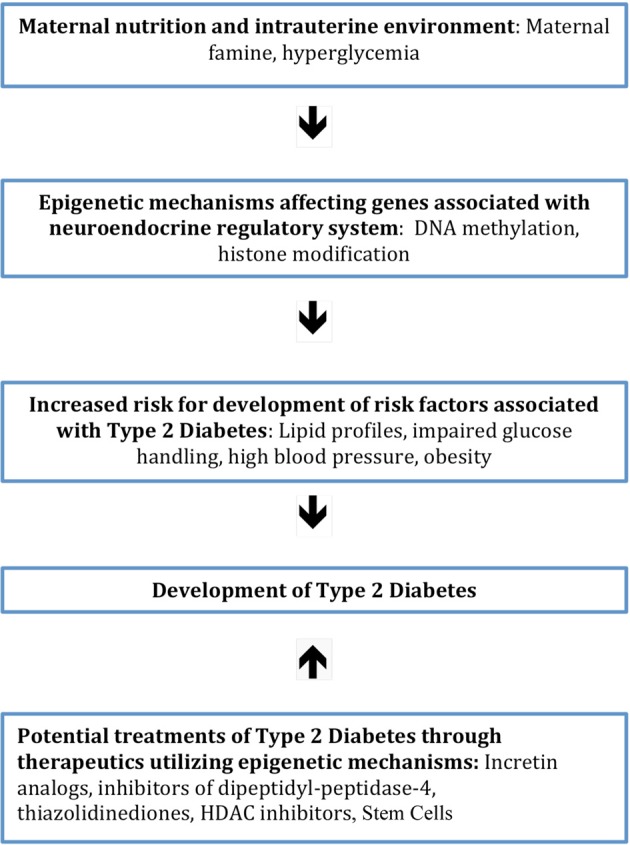
**Diagram showing the sequence of events between factors associated with the intrauterine environment and increased risk for the development of T2DM**. This occurs through the initiation of epigenetic mechanisms that affect genes involving neuroendocrine regulation. Also illustrated is the potential treatment of T2DM with therapeutics that work through epigenetic mechanisms.

The nutritional quality and quantity provided to a developing embryo serves as a strong predictor of the development several risk factors associated with T2DM postnatally. For example, poor nutrient supply within the intrauterine environment is linked to atherogenic lipid profiles, obesity and IGT later in life, all of which are risk factors for developing T2DM (Ravelli et al., 1998; Painter et al., 2006; Wellen et al., 2009; Ludwig and Currie, 2010). Many animal studies have shown that maternal diet causes a permanent change in the methylation of the offspring, with one study demonstrating that mice born to mothers with GDM display hypermethylation and epigenetic downregulation of the *IGF2* and *H19* genes, which is associated with insulin desensitivity (Ding et al., 2012). Differential methylation of the *IGF2* gene, as well as other genes associated with T2DM, has been found to occur in children born to mothers experiencing famine, pointing to epigenetics as the mechanistic bridge connecting prenatal nutrition and the onset of T2DM later in life (Heijmans et al., 2007, 2008; Kaminsky et al., 2009; Tobi et al., 2009). Clearly, maternal nutritional profiles and the intrauterine environment the fetus is exposed to can play a large role in establishing a fetus' risk profile for T2DM and, potentially, other diseases later in life (Figure 2).

The epigenetic profile in the development of T2DM is also being studied primarily *in vitro* as a potential therapeutic aid in the treatment and prevention of metabolic disease. In order for this approach to be fully realized, however, further exploration should be carried out *in vivo* along with clinical trials to determine the efficacy of these molecular interventions to impact patient overall well-being and health. Regardless, the unfolding discovery of epigenetics as a missing link between one's environment and the onset of T2DM is an exciting step forward in our understanding of the multi-factorial development of this disease, one that may, eventually, help reverse the impact of T2DM on global health.

### Conflict of interest statement

The authors declare that the research was conducted in the absence of any commercial or financial relationships that could be construed as a potential conflict of interest.
